# Perinatal Mood and Anxiety Disorder and Reproductive Justice: Examining Unmet Needs for Mental Health and Social Services in a National Cohort

**DOI:** 10.1089/heq.2022.0207

**Published:** 2024-01-24

**Authors:** Tanya Khemet Taiwo, Keisha Goode, P. Mimi Niles, Kathrin Stoll, Nisha Malhotra, Saraswathi Vedam

**Affiliations:** ^1^ Birth Place Lab, Division of Midwifery, Faculty of Medicine, University of British Columbia, Vancouver, British Columbia, Canada.; ^2^ Bastyr University Department of Midwifery, Kenmore, Washington, USA.; ^3^ SUNY Old Westbury, Old Westbury, New York, USA.; ^4^ National Association of Certified Professional Midwives, Keene, New Hampshire, USA.; ^5^ Rtory Meyers College of Nursing, New York University, New York, New York, USA.

**Keywords:** pregnancy, disparities, maternal mental health, person-centered care, reproductive justice, perinatal mood disorders

## Abstract

**Introduction:**

Perinatal Mood and Anxiety Disorders (PMADs) are the most common complications during the perinatal period. There is limited understanding of the gaps between need and provision of comprehensive health services for childbearing people, especially among racialized populations.

**Methods:**

The Giving Voice to Mothers Study (GVtM; *n*=2700), led by a multistakeholder, Steering Council, captured experiences of engaging with perinatal services across the United States, including access, respectful care, and health systems' responsiveness. A patient-designed survey included variables to assess relationships between race, care provider type (midwife or doctor), and needs for psychosocial health services. We calculated summary statistics and tested for significant differences across racialized groups, subsequently reporting odds ratios (ORs) for each group.

**Results:**

Among all respondents, 11% (*n*=274) reported unmet needs for social and mental health services. Indigenous women were three times as likely to have unmet needs for treatment for depression (OR [95% confidence interval, CI]: 3.1 [1.5–6.5]) or mental health counseling (OR [95% CI]: 2.8 [1.5–5.4]), followed by Black women (OR [95% CI]: 1.8 [1.2–2.8] and 2.4 [1.7–3.4]). Odds of postpartum screening for PMAD were significantly lower for Latina women (OR [95% CI]=0.6 [0.4–0.8]). Those with midwife providers were significantly more likely to report screening for anxiety or depression (OR [95% CI]=1.81 [1.45–2.23]) than those with physician providers.

**Discussion:**

We found significant unmet need for mental health screening and treatment in the United States. Our results confirm racial disparities in referrals to social services and highlight differences across provider types. We discuss barriers to the integration of assessments and interventions for PMAD into routine perinatal services.

**Implications:**

We propose incentivizing reimbursement schema for screening and treatment programs; for community-based organizations that provide mental health and social services; and for culture-centered midwife-led perinatal and birth centers. Addressing these gaps is essential to reproductive justice.

## Introduction

In the current discourse on quality of health services, there is belated but heightened awareness of the extreme disparities in maternal mortality.^1,2^ Black and Native American/Alaska Native people experienced higher rates of pregnancy-related deaths than all other groups in the United States, with widening gaps with increasing age and/or higher education.^3^ These deeply concerning statistics do not capture preventable morbidity and mortality associated with psychosocial concerns such as Perinatal Mood and Anxiety Disorder (PMAD). With higher rates of these psychosocial risk factors among women of color, it is critical to understand if and how universal screening and treatment are being offered across populations.^4^ This article focuses on health and safety concerns, service needs, and postpartum screening among a diverse national sample of women who reported on their access to mental health services.

### PMAD: prevalence and impact

PMADs are both the most common complications that women experience during their pregnancy and the most underdiagnosed.^5,6^ One in 10 women suffer from depression every year, and the risk is higher for new mothers and those with very young children.^7,8^ One in five pregnant or postpartum women are diagnosed with a mental health condition.^9^ A systematic review of common anxiety disorders in the perinatal period estimated the prevalence of having one or more anxiety disorders to be 20.7% (95% density interval: 16.7–25.4), with evidence of a slightly greater prevalence during pregnancy than the postpartum period (3.1%).^10^ In fact, although perinatal anxiety is given less attention, these disorders have significant overlap with depression and can often manifest earlier.^11^

About 17% of women will be diagnosed with depression at some point in their lives, and this doubles for those women who are living in poverty.^5^ Black and Hispanic women are at a higher risk for PMADs compared to White women.^12^ People who are uninsured or underinsured, receive Medicaid,^13^ or are in rural settings and/or communities of color are less likely to receive essential mental health services^12^ due to transportation or childcare challenges.^14^ Those with low income are more likely to experience chronic stress, depression, substance abuse, and chronic health conditions; they are also at a higher risk for posttraumatic stress and anxiety.^15^

Finally, in the United States, perinatal depressive disorders are especially high among recent immigrants and ethnic minority groups from non-English-speaking countries.^16^ Refugee and immigrant women face unique challenges in accessing perinatal mental health care, including language barriers, cultural differences, and lack of familiarity with the health care system.^17^

PMAD increase risks to both mother and child. Neurodevelopmental delay, behavioral problems, attachment disorders, depression, and other mood disorders in childhood and adolescence occur more frequently in children of mothers who suffer with major depression.^18^ Anxiety in pregnancy is associated with shorter gestation and has adverse implications for fetal neurodevelopment.^19^ Depressive symptoms during pregnancy are associated with having an infant with low birth weight.^19^

During the perinatal and postpartum periods, women are at a much higher risk for self-harm and suicide.^6,20^ Suicide exceeds hemorrhage and hypertensive disorders as a cause of maternal mortality.^20^ In a recent Center for Disease Control and Prevention (CDC) report compiled from nine maternal mortality review committees, mental health was identified as a leading cause of pregnancy-related death.^21^ The personal circumstances and negative consequences associated with perinatal anxiety and depression are well documented, but less is known about availability, access, uptake, or population-specific prevalence and need for perinatal mental health services.

### Structural and health sytem factors

Any discussion of interventions to improve Indigenous and Black maternal health and well-being must be seated within a reproductive justice framework that addresses social justice and reproductive rights and centers equity and empowerment.^22^ This framework analyzes systems of power, addresses intersecting oppressions, and centers the most marginalized communities.^23–25^ PMAD often co-occur with other challenges to healthy parenting, such as poverty, substance use, and previous trauma, and contribute to cumulative risk.^10^ A reproductive justice approach ensures that all individuals can access all of the resources and support they need to have safe and healthy perinatal experiences, including housing, economic stability, and social services. PMAD can have significant impacts on the well-being of parents and babies from preconception through early parenting,^18^ and addressing behavioral health disorders is therefore an important component of reproductive justice.^26^

There are significant gaps in understanding the drivers and prevalance of PMAD across at-risk populations. Social and structural determinants of health impact not only a person's risk, they also determine subsequent access to perinatal mental health care. Access and quality of screening and treatment are vital factors that are not equitably distributed across communities. Factors such as limited insurance coverage, provider shortages, and regional maldistribution of mental health care facilities can have acute consequences and leave pregnant and postpartum people without a safety net.

Until recently, there was a scarcity of validated person-centered measures of access, uptake, and need for mental health support during the perinatal period, and few datasets capture the unique contexts of care among communities of color. We sought to understand the prevalence of self-identified need for psychosocial support versus rates of actual screening and treatment for PMAD among populations most at risk for adverse mental health and perinatal outcomes using a person-centered survey instrument. The gap between self-identified psychosocial needs and service provision is referenced herein as ***unmet needs***. We examined the experience of adverse psychosocial events during pregnancy, the prevalence of unmet needs, and rates of screening for depression and anxiety among participants in a national survey. We also assessed whether the rates of adverse events, unmet needs, and screening varied by race or ethnicity or who was providing perinatal health services.

## Methods

The Giving Voice to Mothers Study (GVtM) was designed and led by a national, multistakeholder Steering Council, including service users, clinicians, and leaders of community-based perinatal organizations across the United States.^20^ They codeveloped and distributed a survey to capture outcomes and experiences of engaging with perinatal services, including access, respectful care, and health systems' responsiveness. The study was approved by the Ethics Board at University of British Columbia (#H15-01524).

### Survey development

Service users, including those from racialized and marginalized populations, were involved throughout the process, including in defining the survey topics; reviewing items from previous maternity care experience surveys; and suggesting new survey items that captured their lived experiences. Community partners helped to identify service users to participate in content validation of the survey instrument and 55 representative service users rated each item for relevance, importance, and clarity. The online survey tool was forward and backward translated into Spanish and participants had the option of completing the English or Spanish version. The survey took on average 45–60 minutes to complete. Details on survey construction and findings on respectful care have been reported elsewhere.^27^

### Sample and recruitment

All people who had experienced a pregnancy in the United States within 5 years of data collection were eligible to complete the online survey. We worked with 10 community partners to recruit and intentionally oversample participants from communities of color (Black, Indigenous, Asian, Hispanic/Latina) and those who planned a community birth, so that we collected enough data from these previously underrepresented groups.^28^ The community partners included midwifery practices, community health workers, and Non-Governmental Organizations (NGOs) who have explicit mandates to provide perinatal support services to marginalized populations in community settings across the United States (e.g., CommonSense Childbirth, Bold Futures, and National Association to Advance Black Birth).

Community partners helped to identify service users to participate in design and content validation of survey items; designed recruiting materials; distributed the survey through their networks; supported respondents to navigate the online tool; identified the key outcomes for analysis; informed and helped conduct the analyses; and participated in interpreting the results.

Data were collected through snowball and network sampling and partners used community-specific social media package tools (template emails, posts, Facebook ads, etc.) to facilitate this process. Between March 2016 and March 2017, a total of 2915 people from all 50 states responded, with 2700 completing items in all sections of the survey. Denominators for some variables varied because the Steering Council recommended that to maximize participation and minimize harm and/or burden, participants be presented with only four mandatory eligibility or version screening items but otherwise could skip questions. For analyses of relevant variables without a known denominator (i.e., for the “check all that apply” variables), we used a sample size of 2138, that is, the number of participants who answered the last question in the survey, as a proxy for those who completed all sections.

### Study measures

The final survey instrument included 219 and assessing sociodemographics (e.g., age, race, and income), pregnancy, and birth (e.g., number of prenatal appointments, type of prenatal care provider, and mode of birth); and 60 items on experiences of care and health and safety concerns during pregnancy (e.g., concerns about safe housing and neighborhood violence, experiences of depression or financial stress during pregnancy, and need for services, such as housing assistance and maternal mental health well-being after birth).

### Key variables

**FIG. 1.**
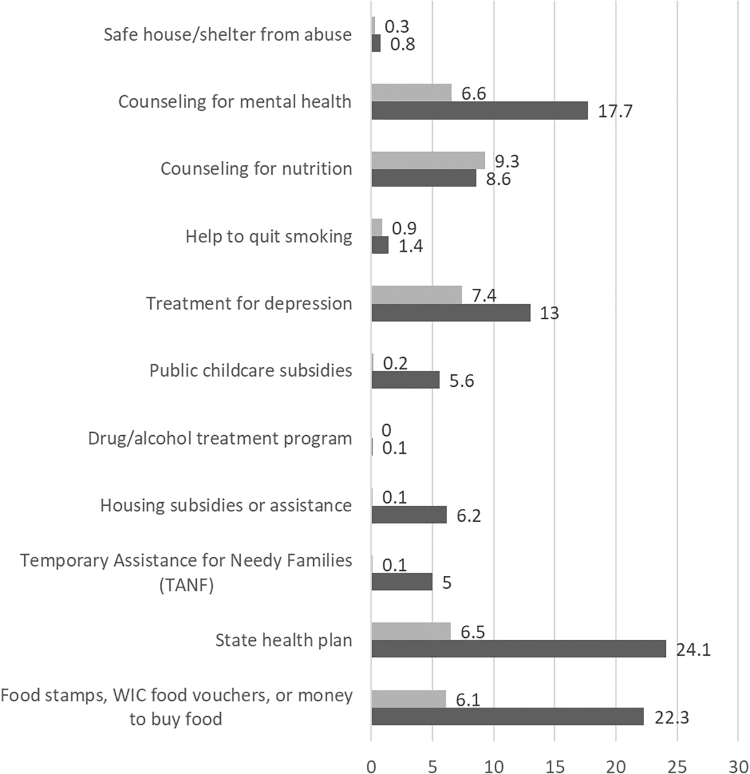
The *dark bar graph* represents support needed, and the *light bar graph* represents the support that care providers helped people access.

In the survey section entitled “Health & Safety,” participants reported on experiences of depression, intimate partner violence, housing instability, drug dependency, and indicators of low socioeconomic status at any point during their pregnancy or the year before. Participants identified social services that they needed during pregnancy: “During your recent pregnancy, did you feel you needed any of the following services?” and whether they had assistance accessing those services: “During your care, did your doctor or midwife help you to get [any of the following services]?” Response options included safe shelter, counseling for mental health, help to quit smoking, and so on (Fig. 1). Participants could check all options that applied.

Respondents self-identified their race/ethnicity by selecting one or more of 13 predefined overall categories, and then had the option to select from several subcategory response options to specify race and ethnicity. They could also select “prefer not to answer” or choose “other” and could provide open-ended comments to delineate their preferred self-identification. We then recoded the responses to the race question into mutually exclusive categories, starting with Black participants. Survey respondents who identified as Black in any of the predefined or open text fields were assigned to that group, regardless of how many other fields they checked.

Next, we coded those who identified as Indigenous in any of the fields as Indigenous (unless they also identified as Black, in which case they were included in the Black category). Next, Asian participants were coded, followed by Hispanic/Latina respondents, other people of color not already assigned to previous categories, and last, those who identified as White. See the GVtM report for more details on the coding approach.^28^

To determine whether key variables differed by model of care, we recoded the prenatal care provider variable, “Which type of caregiver was most directly involved with giving prenatal care?” into two categories: midwife (response options included CNM, CPM, LM, CM, and a midwife but I am not sure of what type) or doctor (response options included: OB-GYN, family doctor, and a doctor but I am not sure of what type).

### Analysis

We report descriptive statistics for all key variables, including the mean number of adverse psychosocial events during pregnancy, the proportion of respondents who experienced each event, and how many reported one or more adverse experience. The mean number of adverse psychosocial events were also stratified by race to examine differences across groups.

We assessed the proportion of people who needed but did not receive help to access psychosocial supports, and then created a binary variable “unmet needs,” which took the value of 1 if the need was not met and 0 if the need was met. This was done for each of the following services: (1) psychosocial support, (2) treatment for depression or anxiety, and (3) counseling for mental health concerns. We also calculated the proportion of people who were asked by care providers (within 8 weeks postpartum) if they experienced (1) physical or verbal abuse during pregnancy or since the birth, (2) symptoms of depression, and/or (3) symptoms of severe stress and/or anxiety. Unmet needs and postpartum screening rates were then stratified by race (Black, Indigenous, Latina, Asian, and White).

Finally, using bivariate logistic regression analysis, we examined the relationship between race/ethnicity and (1) unmet need for four types of support services (housing, health insurance, food stamps, and childcare subsidies) and (2) postpartum screening. In addition to odds ratios (ORs), we present 95% confidence intervals (CIs). SPSS version 26 was used for all descriptive and bivariate analyses. To understand the potential effects of provider type (midwives vs. doctors) on experience of care, we controlled for provider type on the rates of screening for depression and unmet needs. Then we controlled for provider type on the odds of unmet needs as experienced by racialized versus White service users.

## Results

Collaboration with community partners for recruitment efforts yielded a diverse sample (Table 1). Participants from all 50 states completed the survey. Most were U.S. natives (89.6%) and had education beyond high school (96.3%); 28.9% reported household incomes of less than $50,000 per year. Over half of respondents reported structural barriers to optimal health during pregnancy. Depression (21.7%), financial hardship (17.6%), lack of health insurance (16.2%), and inadequate support from family and friends (13.6%) were the most common problems noted (Table 2).

**Table 1.** Sociodemographic Characteristics of Sample (*n*=2700)

Sociodemographic characteristic	***n*** (%)
Age at time of birth	
17–24	132 (5.7)
25–30	736 (31.8)
31–39	1306 (56.4)
40 and older	140 (6.1)
Residence at time of data collection	
New York	778 (28.8)
California	206 (7.6)
Washington	121 (4.5)
Texas	115 (4.3)
Other	1477 (54.8)
Born in the United States	
Yes	2172 (89.6)
No	253 (10.4)
Highest level of education completed	
High school	79 (3.3)
Some college, but no degree	409 (16.9)
College	718 (29.7)
Associate degree	190 (7.8)
Some graduate school, but no degree	176 (7.3)
Graduate degree (e.g., MSc or PhD)	721 (29.8)
Professional degree (e.g., MD or JD)	94 (3.9)
Other	34 (1.4)
Main source of payment for maternity care	
Medicaid/CHIP	365 (13.5)
Private insurance	1371 (50.8)
Out of pocket	514 (19.0)
Other/not sure	450 (16.7)
Total household income before taxes	
$0–19,999	122 (5.8)
$20,000–49,999	485 (23.1)
$50,000–99,999	734 (35.0)
$100,000–159,999	467 (22.3)
$160,000 and over	289 (13.8)
Maternal race	
Black^a^	380 (15.4)
Indigenous/Native American/Native Hawaiian^b^	77 (3.1)
Asian^c^	117 (4.7)
Hispanic/Latina^d^	241 (9.8)
Other person of color (not already allocated)	24 (1.0)
White	1651 (67.0)
Missing values for race variables	210

^a^ Identified as Black or African in any of the race fields.

^b^ Identified as Native American/Native Hawaiian/Indigenous in any of the race fields but not as Black or African.

^c^ Identified as Asian in any of the race fields but not as Black/African/Native American/Native Hawaiian/Indigenous.

^d^ Identified as Hispanic/Latina in any of the race fields but not as Black/African/Native American/Native Hawaiian/Indigenous/Asian.

**Table 2.** At Any Point During Your Pregnancy or the Year Before Your Pregnancy Did You Experience Any of the Following

Adverse experience	***n*** (%)
None of the above	1057 (49.4)
Depression	465 (21.7)
Inability to meet financial obligations	377 (17.6)
Lack of health insurance	347 (16.2)
Not enough support from family or friends	291 (13.6)
Inability to buy enough food	163 (7.6)
Inability to find work	159 (7.4)
Housing instability	109 (5.1)
Smoking (tobacco)	89 (4.2)
Heat or electricity turned off	51 (2.4)
Intimate partner violence	48 (2.2)
Daily alcohol use	40 (1.9)
Police violence, yourself or someone in your family	21 (1.0)
Involvement of child and family services	18 (0.8)

*n*=2138; the percentage does not add to 100 as multiple options could be selected.

### Adverse psychosocial events and unmet needs

The mean number of adverse psychosocial events reported differed by race, with the most reported by Indigenous respondents (1.6), followed by Black respondents (1.3) with Latina, Asian, and White women reporting a mean of less than 1 adverse event (0.8, 0.6, and 0.7), respectively. Figure 1 shows the proportion of people who needed the listed services/support (dark gray) and the proportion who had help from their doctor or midwife to access these services/support (light gray). Of the 11 types of psychosocial support identified as needed by respondents, smoking cessation was the only one where rates of support from care providers closely matched need.

Few respondents received help with accessing other important resources and referrals. These unmet needs included nutrition counseling, intimate partner violence/safe housing, mental health support for depression, drug and alcohol treatment, navigating social safety net programs (public subsidies for housing, childcare, food, insurance, and cash aid), and safe housing/shelter from abuse (Fig. 1).

### Unmet needs and screening stratified by race and provider type

When stratified by race, across all but one area of unmet needs, Black and Indigenous respondents reported the highest percentages (Table 3) of unmet needs. During prenatal care, 147 (5%) respondents reported unmet needs related to treatment for depression, and 239 (9%) did not receive counseling for their mental health concerns.

**Table 3.** Unmet Needs, Including Mental Health and Social Services Stratified By Race

Unmet needs for services	All	Black	Latina	Asian	Indigenous	White
Unmet need: Services for stressors and mental health						
Housing (safe housing subsidies)	126 (8.5)	48 (19.4)	8 (6.0)	4 (6.8)	13 (26.5)	47 (5.0)
State health plan/IHS	335 (21.7)	84 (33.5)	27 (19.6)	9 (14.8)	17 (31.5)	186 (18.8)
Food stamps	330 (21.1)	96 (37.4)	32 (22.9)	9 (14.8)	20 (37.0)	164 (16.3)
TANF/childcare subsidies	160 (10.8)	52 (21.5)	15 (11.3)	4 (6.8)	16 (31.4)	65 (6.8)
Depression	147 (5.4)	33 (13.8)	17 (13.0)	9 (15.0)	10 (21.3)	75 (7.9)
Mental health counseling	239 (8.9)	62 (26.1)	25 (19.1)	13 (22.4)	14 (29.2)	118 (12.5)
Postpartum mental health screening (in the first 8 weeks)						
Physical/verbal abuse	713 (33.9)	467 (33.6)	68 (36.4)	17 (19.5)	31 (49.2)	108 (34.4)
Depression	1647 (78.2)	1119 (80.4)	133 (70.7)	63 (71.6)	54 (85.7)	234 (74.5)
Stress/anxiety	1474 (70.1)	997 (71.6)	126 (67.4)	53 (60.2)	47 (74.6)	210 (66.9)

The percentages do not add to 100 as multiple options could be selected for these questions.

IHS, Indian Health Service; TANF, Temporary Assistance for Needy Families.

Also, in Table 3, we report the rates of screening by race during the postpartum period. Rates of postpartum screening were highest for depression and anxiety, 78.2% and 70.1%, respectively.

In the regression analysis, we found that the odds of reporting unmet needs were greater among Black and Indigenous participants than among those who self-identified as White. In Table 4, we report ORs (and 95% CI) ranging from 2.2 (1.6–2.9) for state health plan information to 4.6 (3.0–7.1) for Black women, and ranging from 2.0 (1.1–3.6) to 6.9 (3.4–13.8) for Indigenous women for the same social support. When disaggregated by race among those who reported unmet needs related to treatment for depression and mental health counseling, Indigenous women were most likely to have significantly more unmet needs (OR [95% CI]: 3.1 [1.5–6.3] and 2.8 [1.5–5.4]), followed by Black women (OR [95% CI]: 1.8 [1.2–2.8] and 2.4 [1.7–3.4]; Table 4) when compared to experiences among White participants.

**Table 4.** Adjusted Odds Ratios for Unmet Needs for Services Stratified By Race

Unmet needs for services	***N***	White	Black	Latina	Asian	Indigenous
Unmet need: Services for stressors and mental health						
Housing (safe housing subsidies)	1450	1	4.6^a^ (3.0–7.1)	1.2 (0.6–2.6)	1.4 (0.5–2.6)	6.9^a^ (3.4–13.8)
State health plan/IHS	1508	1	2.2^a^ (1.6–2.9)	1.05 (0.67–1.64)	0.75 (0.4–1.5)	2.0^a^ (1.1–3.6)
Food stamps	1532	1	3.1^a^ (2.3–4.2)	1.5^a^ (1.0–2.4)	0.9 (0.4–1.9)	3.0^a^ (1.7–5.4)
TANF/childcare subsidies	1454	1	3.7^a^ (2.5–5.6)	1.7 (0.9–3.1)	1.0 (0.4–2.8)	6.3^a^ (3.3–11.9)
Depression	1435	1	1.8^a^ (1.2, 2.8)	1.7 (0.9–3.0)	2.0. (0.9–4.3)	3.1^a^ (1.5–6.5)
Mental health counseling	1427	1	2.4^a^ (1.7–3.4)	1.6^a^ (1.0–2.6)	2.0^a^ (1.0–3.8)	2.8^a^ (1.5–5.4)
Postpartum mental health screening (in the first 8 weeks)						
Physical/verbal abuse	2101	1	1.0 (0.8, 1.3)	1.1 (0.8–1.5)	0.5^a^ (0.3–0.8)	1.9^a^ (1.2–3.2)
Depression	2105	1	0.7^a^ (0.5–0.9)	0.6^a^ (0.4–0.8)	0.6 (0.4–1.0)	1.5 (0.7–3.0)
Stress/anxiety	2104	1	0.8 (0.6–1.1)	0.8 (0.6–1.1)	0.6 (0.4–0.9)	1.2 (0.7–2.1)

Adjusted ORs are reported with 95% CI levels (brackets).

All models are adjusted for care provider type (then list the care provider options in brackets).

^a^ Significant at 5%.

CI, confidence interval; OR, odds ratio.

We also found that Latinas reported the lowest rates of postpartum screening for depression. Indigenous women are significantly more likely than other racialized service users to be assessed for physical and verbal abuse (OR [95% CI]=1.9 [1.2–3.2]) but less likely to be screened for depression than Latina, Asian, and Black respondents. Asian women are significantly less likely to be screened for abuse and anxiety (OR [95% CI]=0.5 [0.3–0.8]), and Latina and Black women are less likely to be screened for depression (OR [95% CI]=0.6 [0.4–0.8] and 0.70 [0.5–0.9]), respectively, than White women (Table 4). When assessed as a whole cohort of racialized service users, in comparison to White women, there is a tendency toward screening Indigenous women and not screening Latina, Asian, and Black respondents.

When we stratified respondents according to who provided their prenatal care, midwifery clients were more likely to report depression screening (81.8% vs. 71.3% of physician patients). Likewise, midwifery clients were more likely to report being assessed for severe stress/anxiety (74.2% vs. 62.7% of physician patients).

Those who received antepartum midwifery services were significantly more likely to report being screened for anxiety, depression, or stress during postpartum visits (OR [95% CI]=1.81 [1.45–2.23]) when compared to those who received care from a physician. Among midwifery patients, Black women were significantly more likely to report unmet needs for mental health counseling and treatment. In addition, both Black (OR [95% CI]=2.35 [1.4–3.9]) and Indigenous women (OR [95% CI]=3.85 [1.3–11]) were more likely to have unmet needs when they reported receiving prenatal care from a physician.

## Discussion

This is the first study to examine experience of perinatal services among underrepresented populations in a national dataset, generated by a patient-oriented instrument. We assessed the lived experience of gaps in health assessment and provision of services during the course of childbearing, in particular, unmet needs for PMAD health services. Our findings show high rates of adverse psychosocial events during the perinatal period among racialized communities and missed opportunities for critical interventions. Care providers did not meet respondents' needs for psychosocial support, screening, or help with navigating social safety net programs. In the postpartum period, most people were screened for depression, stress, and anxiety but significantly fewer reported uptake of mental health services.

All racialized respondents reported less uptake of psychosocial support throughout the perinatal course. When the results were controlled for race, Indigenous and Black women were two to three times more likely to report unmet needs for mental health services. Overall, midwifery patients were more likely to report screening for PMAD than those receiving care from a physician, but their care did not eliminate experiences of unmet needs.

Given the prevalence of PMADs, this analysis offers an understanding of the responsiveness of the health care system to address the mental and social care needs of systematically marginalized people. Our curated selection of these survey items reflects the ways in which services and outcomes are inextricably linked. The lack of prevention, screening, and treatment of PMADs, which are demonstrably higher in systematically marginalized groups, reify the lifecourse impacts of childbearing.

### Unmet need and racialized health disparities

Our findings of differences in rates of screening fall within the context of the significant racial and ethnic disparities in maternal outcomes that persist in the United States and can be explicated by mounting evidence of the role that structural racism plays. For example, we found that Indigenous women were twice as likely to be assessed for physical and verbal abuse as White women. According to an extensive report by Amnesty International, sexual violence against U.S. Indigenous women is seen as normal by statutory legal and health systems, a bias prompting greater screening for these patients as documented in our study.^29^

In addition, we found that among midwifery patients, Black women were significantly more likely to report unmet needs for mental health counseling and treatment, and both Black and Indigenous women were more likely to have unmet needs when receiving prenatal care from physicians. Asian respondents were significantly less likely to be screened for abuse and anxiety, and Latina women were less likely to be screened for depression.

### Implications for practice

The perinatal period presents an ideal time for people with depression and anxiety to be identified and treated. Universal screening for PMAD, risk assessment, and treatment or referral have been recommended by professional bodies and United States Preventative Services Task Force.^6,7,30^ Despite these clear guidelines from professional colleges, it is estimated that 50% of all people with PMAD are never identified.^31^ Most people regard screening as beneficial and indicative of caring by their providers.^32^ People reported that they would discuss their mental health concerns if they believed that their providers were interested, sensitive, and caring^33^ and wanted to know that help was available.^33^

A systematic review by Byatt et al. identified barriers to screening for obstetricians and midwives.^34^ The barriers that discourage the integration of screening and interventions for PMAD include lack of knowledge and skills, their lack of identification as a mental health specialist, and the absence of a systematic referral process.^34^ These factors have led to a lack of integration of the treatment of mood disorders within the maternity care setting.^34^

Henke et al. identified six barriers among physicians to treating patients with depression: difficulty diagnosing depression, patient resistance, fragmented mental health system, insurance coverage, lack of expertise, and competing demands and other responsibilities as a primary care provider.^35^ Additional barriers to treatment of PMAD identified by providers include lack of time, limited knowledge of available resources, and the perceived reluctance of their patients to engage in depression treatment.^34^ Some primary care physicians identified inclusion of care managers, mental health integration, and patient education as potentially helpful interventions, although these did not address the systemic barriers of the fragmented mental health system and insurance coverage limitations.^35^

### Health equity implications

The reproductive care system in the United States reflects the overall health of the society. That is, the ways in which we do—and do not—support emerging families are a strong indicator of the nation's values and overall well-being. While the focus of this article is on the role and responsibility of perinatal health care providers, we also observe a public health responsibility. The Institute for Medicaid Innovation (2018) identified key research, clinical, and policy opportunities to improve provider screening guidelines and treatment for maternal depression and anxiety.^36^ Vital to public health are campaigns that raise awareness, educate, and identify standard interventions to support mental health across populations. These standards are more feasible in single payor national insurance schema that facilitate easier access to care at little or no cost.^5^ Additional services provided by integrated systems include perinatal counseling, physical therapy both before and after delivery, and in-home support.^5^

Notably, while midwives in this sample were more likely to screen for PMAD, it was not sufficient to close the gap of unmet needs. Recent publications have cited the adverse impacts of protocols, time limits, and definitions of scope and model of care that pre-exist in institutional settings and hinder the ability of midwives to provide relationship-based care.^37–39^ Relationship-based and continuity models of care are associated with a greater sense of well-being and trust among recipients of care.^40–42^ In addition, care provided in culture-centered settings focuses on health promotion; needs assessments for social, cultural, and mental health support; and enough time to build the relationships and trust necessary to ensure early detection, prevention, and anticipatory guidance or referrals.^38,43^

We urge universal PMAD screening for every pregnant person because we believe it to be essential to holistic care; yet we situate this discussion within a historical context of medical racism in the United States. Historically, Black and Indigenous bodies have been socially marked by legacies of colonization and slavery, Jim Crow laws, racist research, and social policies that institutionalized and constitute daily forms of harmful experiences.^44–49^ Perceptions and experiences of safety and trust are essential to quality care, including effective, trauma-informed health assessment by providers and disclosure by victims. Researchers have found that cumulative trauma exposure experienced by women of color is estimated to be between 51% and 69%.^50^

Our findings align with an analysis of disparities in mistreatment during pregnancy and birth among Giving Voice to Mothers respondents.^27^ In the current analysis, Black and Indigenous respondents had the highest mean rates of social determinants of adverse psychosocial health outcomes during their pregnancy. We already know that posttraumatic stress disorder (PTSD) is more prevalent in Black women than any other racial-ethnic group.^50^ Further, in a sample of 150 Black women who had given birth, 60% of women reported cumulative trauma and partner conflict, and nearly one fourth of these women screened were at a positive risk for PTSD.^51^ The negligence in screening women for PMAD must not be another point of stress exposure.

The prevalence and risks associated with PMAD compel us to call for development of an effective multidisciplinary approach to stimulate clinical, program, and system-level change. Health systems must be held accountable, and, accordingly, the reimbursement schema for screening and treatment must be expanded. Greater investment in community-based health workers who are already extending social networks to support at-risk individuals as well as community-based and culture-centered care at birth centers may offer solutions that stimulate both early detection and greater uptake in historically marginalized communities.^38,43^

### Strengths and limitations

The US-based GVtM study is the first national study to examine maternity care using patient-designed measures on experience of care and patient-reported data on access to health services, including the gaps between perceived need and actual provision of mental health services. The convenience sampling frame prevents us from generalizing findings to all childbearing people in the United States. However, the geographic distribution of responses suggests that our findings are resonant across the country. In addition, our deliberate community-based participatory sampling strategies resulted in somewhat higher proportions than the U.S. Census data on distribution for Black and Indigenous service users (Black 15.4% vs. 14.2%; Indigenous 3% vs. 1%), providing unprecedented data on communities most at risk for adverse perinatal health outcomes.

## Conclusion

For many families, pregnancy, childbirth, and postpartum care provide a gateway to lifelong health, if the health care system offers preventative and harm-reducing approaches. Perinatal care providers play a pivotal role in supporting physical and psychosocial wellness throughout the childbearing cycle. Our research found significant unmet need for psychosocial support services, including mental health screening and treatment, in a large, diverse U.S. sample. Further, the unmet need was greater among Indigenous and Black women. We propose incentives for greater adherence to national screening guidelines, reimbursement for screening and treatment programs, community mental health workers, and culture-centered midwife-led birth centers. PMAD screening is an important reproductive justice strategy for effective health care reform.

## Data Access Statement

Research data supporting this publication are available from the UBC Abacus Dataverse Network: https://dx.doi.org/10.14288/1.0363296

## Abbreviations Used

CDC: Center for Disease Control and Prevention
GVtM: Giving Voice to Mothers Study
IHS: Indian Health Service
PMAD: Perinatal Mood and Anxiety Disorder
TANF: Temporary Assistance for Needy Families
